# Infrared radiation alleviates damage to epidermal melanocytes induced by ultraviolet irradiation: potential for improving the efficacy of phototherapy for vitiligo lesions

**DOI:** 10.3389/fbioe.2026.1852981

**Published:** 2026-08-21

**Authors:** Hua-Ye Bao, Rong Jin, Fuyue Wu, Xiaomin Shao, Zheng Lin Tan, Aie Xu

**Affiliations:** 1 Department of Dermatology, Hangzhou Third People’s Hospital, Hangzhou, China; 2 ReMed Regenerative Medicine Clinical Application Institute, Shanghai, China; 3 Yunnan Characteristic Plant Extraction Laboratory Co., Ltd., Yunnan, China

**Keywords:** infrared, melanocyte, phototherapy, ultraviolet, vitiligo

## Abstract

Ultraviolet (UV) phototherapy has long been considered an effective method for treating skin pigmentary disorders. However, the efficacy of phototherapy is significantly lower than surgical approaches. This might be due to cell death caused by ultraviolet irradiation. This finding contradicts the common understanding of the role of ultraviolet irradiation in promoting the proliferation and migration of melanocytes and improving the efficacy of phototherapy in the treatment of pigmentary disorders, e.g., vitiligo, is an urgent task. In this study, we investigated the combinatorial effect of infrared (IR) radiation and UV irradiation on human epidermal melanocytes. Changes in gene expression were investigated by transcriptomic analysis, and the related mechanisms were analyzed. We discovered that IR protects epidermal melanocytes against cell death induced by UV irradiation and provides the benefit of enhanced melanin synthesis when combined with UV irradiation. We then evaluated the protective effect of IR against DNA damage and its effect on melanocyte proliferation. The results indicated that IR radiation could promote melanocyte proliferation while maintaining the melanin biosynthesis-promoting effect induced by ultraviolet irradiation. This is particularly useful for the treatment of vitiligo as it protects melanocytes against cell death while improving melanin synthesis, which could contribute to the repigmentation of vitiligo lesions. In conclusion, this study has shown that IR potentially protects normal melanocytes against damage induced by ultraviolet irradiation while enhancing the effect of ultraviolet irradiation on promoting melanin biosynthesis.

## 1 Introduction

Vitiligo is an autoimmune disease that results in the progressive loss of melanocytes and the formation of depigmented lesions on the skin (Passeron, 2020). The global lifetime prevalence of vitiligo is 0.36% in general adult population and 0.24% in general child population. The prevalence in Central Europe and South Asia is 0.52%, which is higher than that in other regions (Akl et al., 2024). While subjective symptoms of vitiligo are rarely reported, vitiligo usually persists for years or throughout the lifetime of patients, sometimes affecting exposed skin, which can be disfiguring and often results in depression. Vitiligo places a great burden on both patients and their families, reducing their quality of life (Narayan et al., 2021).

The pathogenesis of vitiligo involves a variety of factors, including autoreactive T cells, genetic predisposition, environmental influences, and melanocyte stress. Yet, none of these factors explain the exact cause of vitiligo (Boniface et al., 2016), rendering the treatment of vitiligo ineffective. Currently, surgical approaches and non-surgical approaches are available as treatment options for vitiligo (Pigmentary Disorder Group, 2024; Combination of Traditional and Western Medicine Dermatology, Research Center for Vitiligo and Chinese Society of Dermatology, Committee on Pigmentation Disorders, China Dermatologist Association, 2024). Non-surgical approaches include the application of corticosteroids, immune modulators, e.g., Janus kinase inhibitors, and phototherapy; surgical approaches involve the transplantation of non-cultured epidermal suspension, autologous cultured melanocytes, or epithelial cell suspensions.

Among the various approaches for vitiligo treatment, phototherapy with narrow-band ultraviolet B (NB-UVB) has been considered the gold standard and mainstay of vitiligo treatment for decades (Bae et al., 2017). While the mechanisms remain largely ambiguous, it is generally considered that NB-UVB can induce tyrosinase activity, followed by melanin synthesis, in addition to promoting melanocyte proliferation and migration (Zubair and Hamzavi, 2020). However, it is important to note that the efficacy of NB-UVB phototherapy is inadequate. Compared with surgical approaches, which have demonstrated a treatment response rate of 81.01% for patients achieving ≥50% repigmentation (Ju et al., 2021), the rate for NB-UVB is approximately 37.4% after 6 months of treatment (Bae et al., 2017). While NB-UVB is generally regarded as a factor that can induce the proliferation and maturation of melanocytes, what limits the repigmentation efficacy of NB-UVB remains uncertain.

Recently, a study suggested that irradiation with UVB could induce melanocyte cell death (Xu et al., 2025). While core ferroptosis-related genes were not upregulated, RAS, repressor activator protein 1, phosphoinositide-3-kinase–AKT, mitogen-activated protein kinase signaling pathways, and iron metabolism-related genes were activated, suggesting that melanocyte damage was potentially induced by ferroptosis after UVB exposure. Xie et al. (2022) showed that 300 mJ cm^-2^ induces melanocyte and keratinocyte apoptosis through ROS generation, suggesting that UVB exposure not only induces ferroptosis but also increases ROS production, which further induces melanocyte cell death. While DNA damage in keratinocytes might activate p53, which, in turn, upregulates pro-opiomelanocortin and promotes the release of α-melanocyte-stimulating hormone to promote growth and maturation of melanocytes, this function might be disrupted in vitiligous lesions, where epidermal keratinocytes are affected by ROS (Xie et al., 2022). Not to mention that the DNA of melanocytes might be damaged by UVB. This is further supported by another study on melanocytes, which suggested that exposure to 40 mJ cm^-2^ UVB, which is approximately 0.013–0.026-fold of the maximum of clinical dose (Mohammad et al., 2017), could lead to epidermal melanocyte cell death and attenuate proliferation (Wu et al., 2026). These observations contradict the common understanding that NB-UVB can promote the proliferation and maturation of melanocytes. The key to improving efficacy of NB-UVB phototherapy for vitiligo might lie in protecting melanocytes against cell death after NB-UVB phototherapy so that they can proliferate and mature.

On the other hand, some studies have suggested that infrared (IR) radiation could provide a photoprotective effect on dermal cells, particularly dermal fibroblasts, by reducing phototoxicity of UVA (280–320 nm) and UVB (320–400 nm) (Menezes et al., 1998). The protective effect of IR can last for 24 h. However, this study employed a lamp with an emission spectrum ranging 400–2000 nm, covering the spectra of visible light (400–750 nm) and IR (750–2000 nm). Therefore, the effect of visible light through opsins cannot be excluded. The protective mechanism of IR irradiation in normal melanocytes remains uncertain. Nevertheless, these studies suggest that IR radiation can counteract the negative effects of UVB irradiation, and we propose that the potential photoprotective effect of IR against UVB could improve the efficacy of phototherapy for vitiligo.

Based on the results obtained from previous studies, the aim of this study is to investigate the effect of combined IR and UVB irradiation on human epidermal melanocytes. Considering its potential application in improving the efficacy of NB-UVB phototherapy, primary human epidermal melanocytes are used. After irradiation, the transcriptomes of the cells were analyzed. DNA damage and proliferation of cells after irradiation were also evaluated.

## 2 Methods

### 2.1 Materials

Levo-dihydroxyphenylalanine (*L*-DOPA) was obtained from Sigma (People Republic of China); goat antibody against human microphthalmia-associated transcription factor (MITF), NorthernLights^TM^ NL557-conjugated donkey antibody against goat immunoglobulin G (IgG), and NorthernLights^TM^ NL557-conjugated donkey antibody against rabbit IgG were obtained from R&D Systems (Minneapolis, United States); rabbit antibody against human tyrosinase related protein 1 (TYRP1) was obtained from Novus Bio (Colorado, United States); and Cell Counting Kit-8 (CCK8) and γ-H2AX assay kit were obtained from Beyotime (Shanghai, People Republic of China).

### 2.2 Isolation of epidermal melanocytes

Human epidermal melanocytes were isolated using a protocol modified from a previous study (Goff et al., 2023). In brief, sterilized human skin was treated with 10 mg ml^-1^ dispase II (Gibco, Japan) overnight at 4 °C to separate the epidermis from the dermis. The epidermis was digested with 0.25% trypsin-ethylenediaminetetraacetic acid solution (Gibco, New York, United States). Then, the reaction was inhibited, and the remaining tissue was removed by filtration through a 40 μm cell strainer. The filtrate was pelleted by centrifugation at 800 × g for 5 min, followed by resuspension in epidermal melanocyte culture medium (containing fetal bovine serum and bovine pituitary extract) and seeding in a cell culture dish. The cells were cultured in an incubator at 37 °C with 5.0% CO_2_.

### 2.3 *L*-DOPA staining

After passaging, the cells were cultured on poly-L-lysine-coated slides to approximately 60%–80% confluency. The slides were retrieved, rinsed with Dulbecco’s phosphate-buffered saline (D-PBS), and fixed with 4% paraformaldehyde. The cells were rinsed twice before incubation with 0.1% *L*-DOPA solution at 37 °C for 4 h. The stained cells were observed under a bright-field microscope.

### 2.4 Immunocytochemistry

The fixed cells were blocked and incubated with primary and secondary antibodies according to the manufacturer’s instructions. Rabbit antibody against human TYRP1 and goat antibody against human MITF were used as primary antibodies. NorthernLights^TM^ NL557-conjugated donkey antibody against goat IgG and NorthernLights^TM^ NL557-conjugated donkey antibody against rabbit IgG were used as secondary antibodies. A no-primary-antibody-control was prepared by incubating the samples with D-PBS instead of the primary antibodies. Hoechst 33258 was used as a counterstain. The cells were observed under a fluorescence microscope (IX73, Olympus, Tokyo, Japan) using the no-primary-antibody control for background correction.

### 2.5 Transcriptomic analysis of epidermal melanocytes stimulated with various light sources

After epidermal melanocytes were cultured to 80% confluency, the cells were rinsed with D-PBS, and the medium was replaced with D-PBS. The cells were irradiated with 60 J cm^-2^ of 1,000–2,400 nm IR (IR; YYIU01Y, Shenyang Wangli Biotechnology Co., Ltd., Shenyang, China), 0.04 J cm^-2^ of 312 nm UV (UV; Scientz03-II, Ningbo Scientz Biotechnology Co., Ltd., Ningbo, China), or 60 J cm^-2^ of 1,000–2,400 nm IR immediately followed by 0.04 J cm^-2^ 312 nm UV (IR + UV). To simulate actual clinical application, the cells were maintained on a 37 °C heater while receiving IR radiation so that the experiment could account for the effect of heat. A thermometer was placed beside the cells, and changes in temperature were monitored. No change in temperature was recorded.

After stimulation, D-PBS was replaced with epidermal melanocyte culture medium, and the cells were incubated in an incubator at 37 °C with 5.0% CO_2_ for 24 h prior to RNA extraction. Total RNA was isolated using TRIeasy® Total RNA Extraction Reagent (Yeasen Biotechnology, Shanghai, People Republic of China). The total RNA extracts were then snap-frozen and stored at −80 °C until further processing.

Before library construction, RNA purity was analyzed using a microphotospectrometer (NanoDrop 2000, Thermo Scientific, United States), and RNA integrity was analyzed using a bioanalyzer (Agilent 2100 Bioanalyzer, Agilent Technologies, California, United States). The libraries were then constructed using the VAHTS Universal V10 RNA-seq Library Prep Kit (Premixed Version) according to the manufacturer’s instructions. Transcriptome sequencing was performed by Shanghai OE Biotech Co. Ltd. After library construction, the products were sequenced on an Illumina NovaSeq X Plus platform, generating 150 bp paired-end reads. Approximately 33.67 million raw reads were generated for each sample. Raw reads in FASTQ format were processed using fastp (Chen et al., 2018), and low-quality reads were removed to obtain clean reads. Approximately 31.50 million clean reads were obtained per sample. The clean reads were mapped to the reference genome (GRch38. p13) using HISAT2 (Kim et al., 2015). Further analyses were conducted using R (v4.5.1).

### 2.6 Detection of γ-H2AX in epidermal melanocytes stimulated with various light sources

Epidermal melanocytes were irradiated either with IR, UV, or IR + UV as described in the previous section. Then, the D-PBS was replaced with epidermal melanocyte culture medium, and the cells were incubated at 37 °C with 5.0% CO_2_ for 1 h. The cells were then retrieved, rinsed, and fixed with 4% paraformaldehyde, and the expression of γ-H2AX was measured by immunofluorescence staining method using a γ-H2AX assay kit according to the manufacturer’s instructions. Hoechst 33258 was used as a counterstain. The cells were observed under a fluorescence microscope (IX73, Olympus, Tokyo, Japan), using no-primary-antibody control for background correction. A minimum of 10 cells per frame was used to quantify the signal intensity.

### 2.7 Proliferation of epidermal melanocytes stimulated with various light sources

After stimulation with various light sources as described in the previous section, D-PBS was replaced with epidermal melanocyte culture medium supplemented with CCK8 assay reagent according to the manufacturer’s instruction. Absorbance at 450 nm was measured using absorbance at 650 nm as the reference at 2 h, 4 h, 8 h, 24 h, and 48 h. The relative proliferation rate of melanocytes was analyzed by comparing the proliferative activity of melanocytes at two specific time points.

## 3 Results

### 3.1 Morphological changes in human epidermal melanocytes after IR and UV treatment

Human epidermal melanocytes were isolated from human skin and characterized by their morphology, *L*-DOPA staining, and immunofluorescence staining (Supplementary Figure S1). The cells exhibited bipolar, tripolar, and polydendritic morphologies, which are consistent with the morphology of melanocytes (Supplementary Figures S1a,b). Under a bright-field microscope, a brownish hue indicating the presence of melanin pigment was observed (Supplementary Figure S1a), with strong refraction at the cell edges under a phase-contrast microscope (Supplementary Figure S1b). These optical characteristics resembled those of epidermal melanocytes. These cells were *L*-DOPA^+^ cells (Supplementary Figure S1c), indicating their capacity for melanin biosynthesis. They also expressed TYRP1 and MITF, both of which are markers of human epidermal melanocytes (Supplementary Figures S1d,e).

**FIGURE 1:**
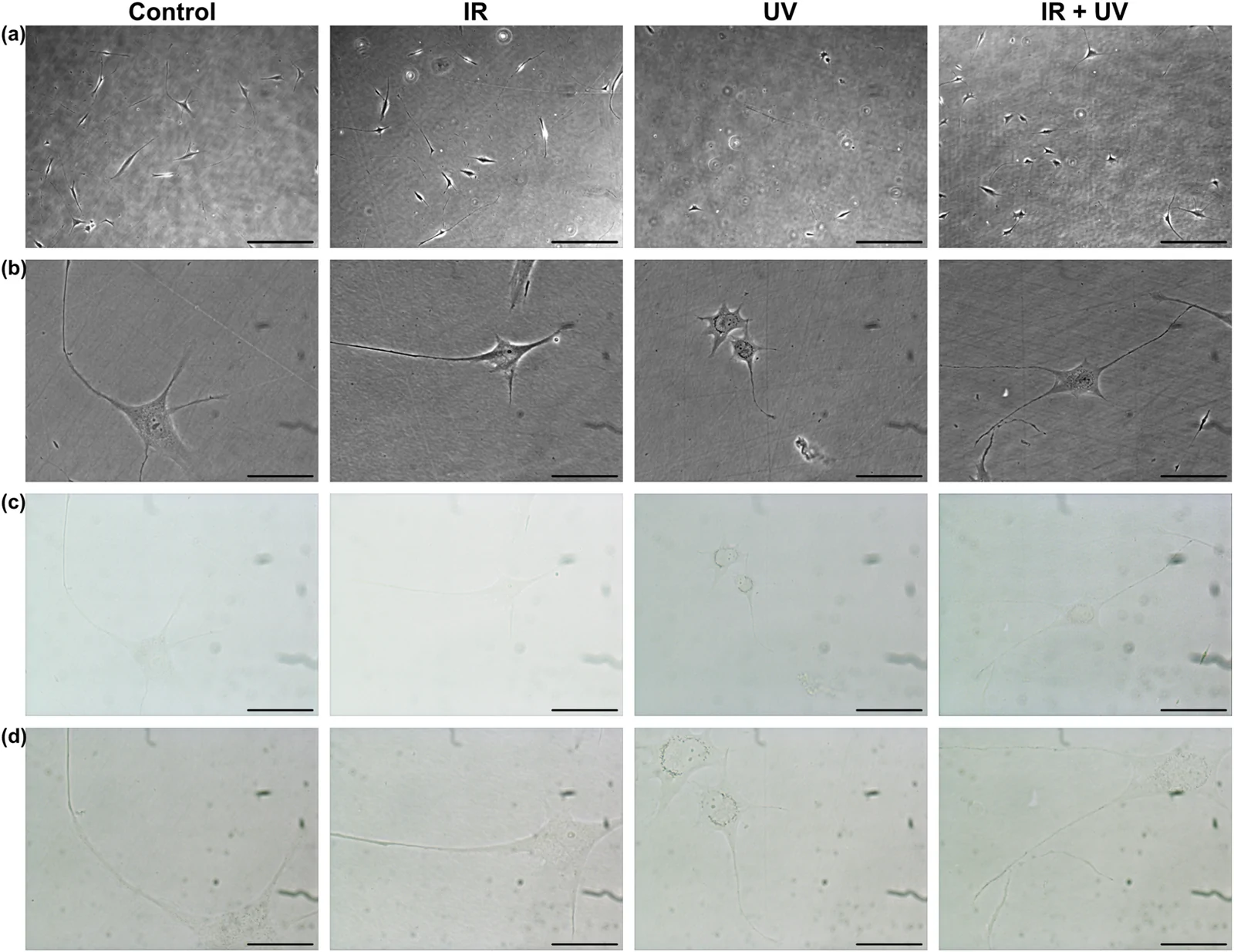
Micrographs of epidermal melanocytes after various treatments. **(a)** Phase contrast micrographs of epidermal melanocytes under ×4 magnification. The scale bars are 500 μm. **(b)** Phase contrast micrographs of epidermal melanocytes under ×20 magnification. The scale bars are 100 μm. **(c)** Bright-field micrographs of epidermal melanocytes under ×20 magnification. The scale bars are 100 μm. **(d)** Bright-field micrographs of epidermal melanocytes under ×40 magnification. The scale bars are 100 μm.

Changes in human epidermal melanocytes at 24 h after UV and IR irradiation were also observed (Figure 1). The number of melanocytes after UV irradiation was lower than in the other groups, suggesting potential cell death and growth arrest of epidermal melanocytes after UV irradiation (Figure 1a). Enlarged images of melanocytes from each group showed that dark melanosomes accumulated in epidermal melanocytes after UV or IR + UV treatments (Figures 1b,c), suggesting potential *de novo* synthesis of melanin in melanocytes after UV irradiation. While there was no sign of melanosome accumulation in the control and IR treatment group, melanosome transfer could be observed, particularly in the dendrites (Figure 1d), indicating that there were no functional changes in melanocytes after IR treatment.

### 3.2 Transcriptomic analysis of epidermal melanocytes after IR and UV irradiation

**FIGURE 2:**
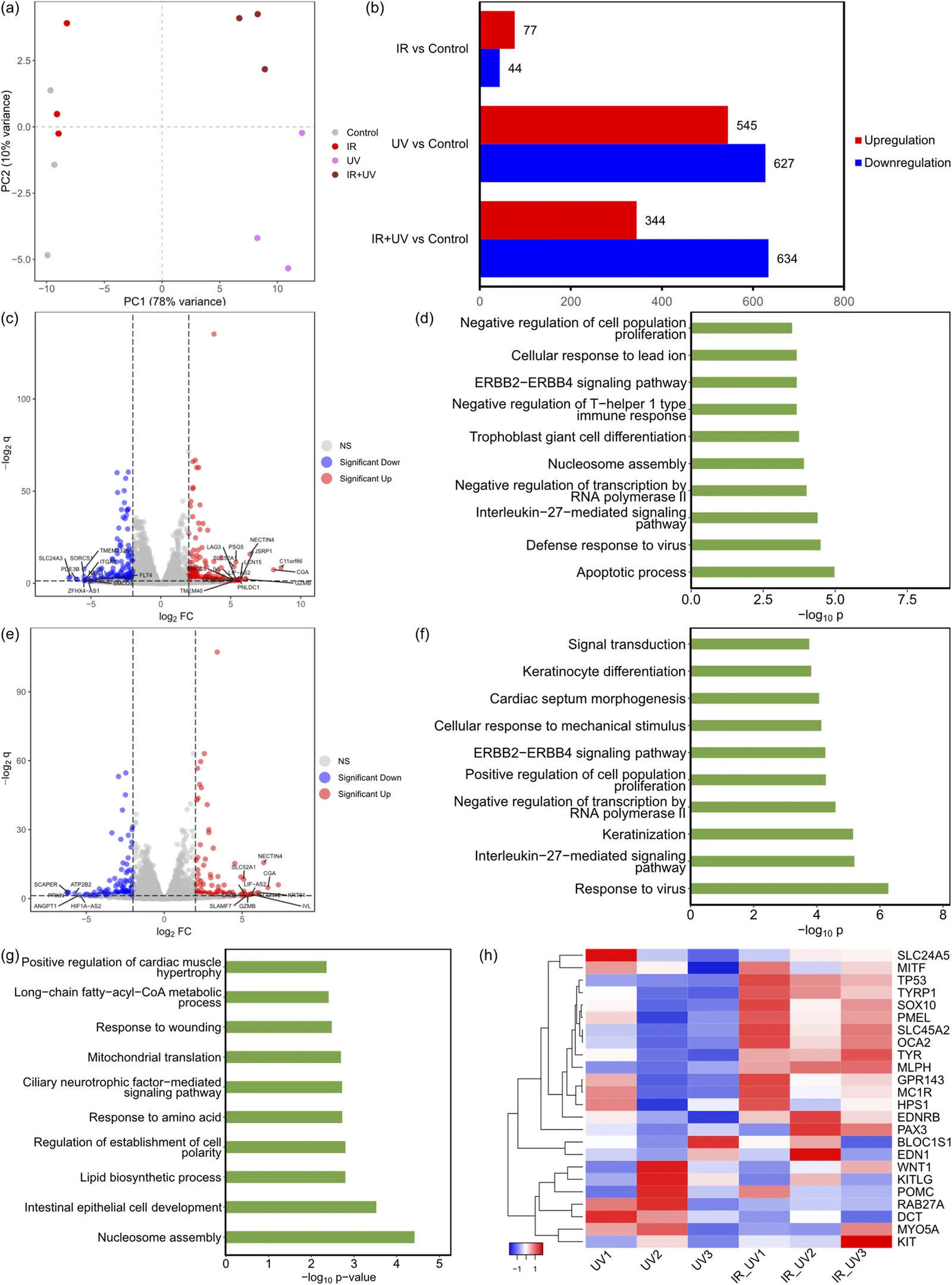
Transcriptomic analysis of human epidermal melanocytes after IR, UV, and IR + UV irradiation. **(a)** PCA of the sequencing data of epidermal melanocytes. **(b)** Number of genes regulated after various treatments relative to the control. **(c)** Volcano plot of sequencing data of UV-irradiated melanocytes against control and **(d)** GO enrichment of upregulated genes. **(e)** Volcano plot of sequencing data of IR + UV-irradiated melanocytes against control and **(f)** GO enrichment of upregulated genes. **(g)** GO enrichment of downregulated genes of IR + UV vs. UV. **(h)** Expression heatmap of genes related to melanin biosynthesis.

To better understand the effects of UV and IR irradiation on human epidermal melanocytes, RNA was extracted from cells treated with IR, UV, and IR + UV were sequenced. Principal component analysis (PCA) of the data suggested that UV irradiation altered the expression profile of the cells, whereas IR irradiation potentially induced the fewest changes (Figure 2a) as the IR group was located closest to the control group.

The numbers of upregulated and downregulated genes in each treatment group compared with the control were analyzed. The numbers of genes upregulated and downregulated in melanocytes after IR irradiation were 77 and 44 genes, respectively (Figure 2b). While no increase in temperature was recorded by the thermometer, it is possible that IR could cause vibration of water molecules, which, in turn, generates heat and upregulates genes related to the heat-induced response. Thus, we have evaluated the expression of genes related to heat-induced response in our data. The result indicated that IR did not alter the expression of genes related to the heat-induced response (Supplementary Figure S2). The number of genes upregulated by UV irradiation was the highest, while the number of genes downregulated by UV irradiation was almost similar to that in the IR + UV group (Figure 2b).

Analysis of gene expression using volcano plots (Figures 2c,e) and enrichment analysis of the upregulated genes in UV-irradiated melanocytes and IR + UV-irradiated melanocytes compared with the control suggested that cell proliferation was negatively regulated in UV-irradiated samples, while being positively regulated in IR + UV-irradiated samples (Figures 2d,f). Furthermore, genes related to apoptotic process were also enriched in UV-irradiated melanocytes. On the other hand, genes related to the ERBB2–ERBB4 signaling pathway, which is associated with melanogenesis, were enriched in both groups, suggesting that UV irradiation could promote melanin biosynthesis.

Comparing the data obtained from IR + UV to UV showed that genes related to the lipid biosynthetic process and the long-chain fatty acyl-CoA metabolic process were downregulated (Figure 2g). Genes, e.g., acyl-CoA synthetase long-chain family member 4, rapamycin-insensitive companion of mammalian target of rapamycin, and far-red-impaired response 1, were enriched in UV response relative to IR + UV. On the other hand, genes in semaphorin receptor binding were enriched in IR + UV, which, through this activity, increased the activity of melanin biosynthesis of the IR + UV group relative to UV (Figure 2h).

The results suggested that while UV irradiation could increase melanin biosynthesis, it also induces cell death in epidermal melanocytes by promoting apoptosis. Pre-treatment with IR could protect epidermal melanocytes against apoptosis while enhancing melanin biosynthesis.

### 3.3 IR protect melanocytes against UV-induced DNA damage

**FIGURE 3:**
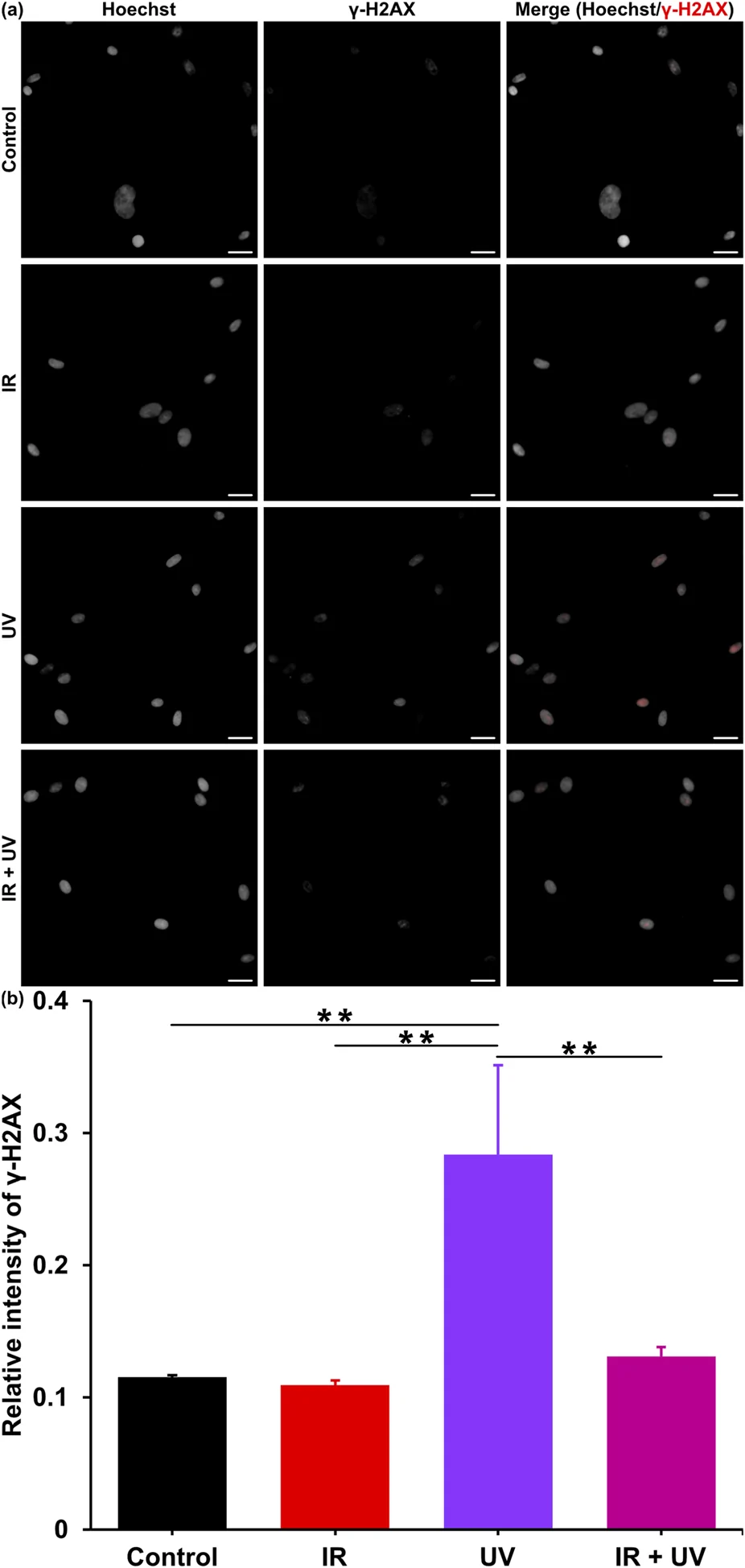
DNA damage induced by irradiation. **(a)** Representative fluorescence photograph of the nucleus of epidermal melanocytes stained with γ-H2AX. The scale bars are 20 μm. **(b)** Relative signal intensity of γ-H2AX against Hoechst. Each plot represents three biological replicates. Data are presented as the mean ± standard deviation. Statistical significance was evaluated using ANOVA with post-hoc Tukey HSD. *p < 0.05, **p < 0.01.

As discussed in the previous section, UV irradiation negatively regulated cell proliferation and induced apoptosis. A major cause of melanocyte apoptosis was DNA fragmentation induced by UV irradiation. After IR, UV, and IR + UV irradiation, the cells were stained with an antibody against γ-H2AX and Hoechst. While UV irradiation could induce DNA fragmentation, as indicated by the signal from γ-H2AX, pre-treatment with IR irradiation could protect the DNA of epidermal melanocytes against fragmentation (Figure 3A). The signal intensity of γ-H2AX was measured against Hoechst, and statistical analysis was conducted (Figure 3b). IR irradiation reduced UV-induced DNA fragmentation to a level similar to that of the control, suggesting the protective effect of IR.

### 3.4 IR recovered the proliferative capability of epidermal melanocytes

**FIGURE 4:**
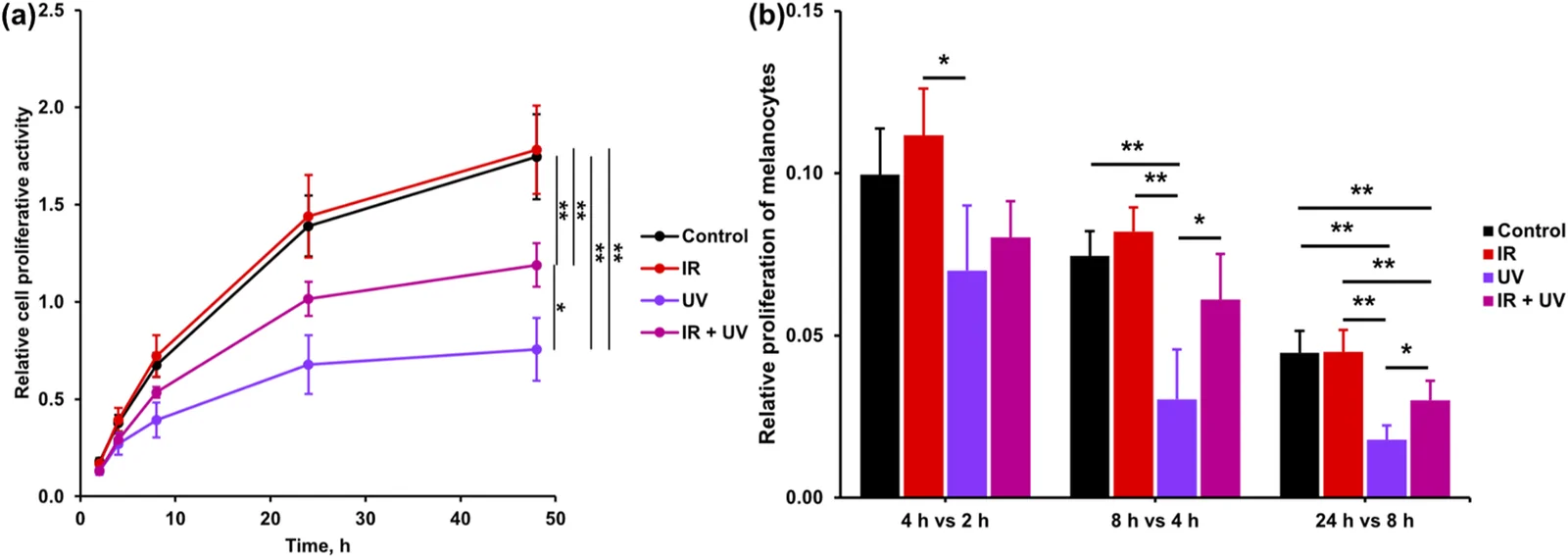
Growth curve of epidermal melanocytes after irradiation. **(a)** Curve of relative proliferation of epidermal melanocytes after irradiation. Each data point represents five biological replicates. The plot represents the mean ± standard deviation. Statistical significance was evaluated using ANOVA with post-hoc Tukey HSD. *p < 0.05, **p < 0.01. **(b)** Rate of proliferation of epidermal melanocytes after irradiation. Each data point represents five biological replicates. The plot represents the mean ± standard deviation. Statistical significance was evaluated using ANOVA with post-hoc Tukey HSD. *p < 0.05, **p < 0.01.

The proliferative activity of epidermal melanocytes was also evaluated (Figure 4). The curve of relative cell proliferative activity over time suggested that UV irradiation attenuated the proliferation of epidermal melanocytes (Figure 4a), which was consistent with the data obtained from transcriptomic analysis. In contrast, IR irradiation positively regulated cell proliferation, which counteracted the effects of UV irradiation. Analysis of the proliferation rate of melanocytes after irradiation suggested that IR irradiation increased the proliferation rate of melanocytes, with or without UV irradiation (Figure 4b).

## 4 Discussion

NB-UVB phototherapy is a double-edged sword in the treatment of vitiligo (Valejo Coelho and Apetato, 2016). Phototherapy induces melanocyte maturation (Zubair and Hamzavi, 2020); on the other hand, it results in cell death, which decreases the number of melanocytes in the skin (Xu et al., 2025). This contradiction potentially limits the efficacy of phototherapy. Protecting melanocytes against cell death after NB-UVB irradiation is key to improving the efficacy of phototherapy.

In this study, we have shown that IR irradiation prior to UV irradiation could protect melanocytes against UV-induced cell death while promoting melanin biosynthesis via the extracellular signal-regulated kinase and phosphoinositide-3-kinase–AKT pathways (Figures 2c–f). IR irradiation alleviates the apoptotic effects of UV irradiation and promotes cell proliferation. Furthermore, by comparing IR + UV to UV, we have found that genes, for instance, acyl-CoA synthetase long-chain family member 4 and rapamycin-insensitive companion of mammalian target of rapamycin, were enriched in UV. These genes are related to ferroptosis (Huang et al., 2024) and UV stress response (Back et al., 2012). Genes related to mitochondrial translation and lipid biosynthesis were also enriched in UV treatment compared with IR + UV treatment, and they are associated with stress responses. By reducing mitochondrial translation, IR reduced energy consumption in cells, prevented misfolding of protein and accumulation of toxin, and promoted metabolic reprogramming, thereby protecting epidermal melanocytes against cell death resulting from UV treatment; by reducing lipid biosynthesis, IR reduced the expression of genes related to general protein translation and activated the central carbon metabolism pathway and lipid droplet generation pathway, which, in turn, protected epidermal melanocytes against stress. In addition, DNAH2, which function to facilitate homologous recombination repair, genes related to NAD biosynthesis, and genes involved in the positive regulation of PI3K signal transduction were upregulated in IR + UV treatment. The regulation of these genes helps protect melanocytes against energy loss, enhances DNA repair and melanin synthesis, and further promotes melanocyte proliferation. Further analysis of the molecular functions of genes enriched in IR + UV treatment revealed enrichment of genes related to semaphorin receptor binding, including SEMA3A and SEMA4D. Semaphorin is a potential mediator of melanocyte–neuron interactions, and its expression is associated with migration, survival, cytoskeletal remodeling, and melanin biosynthesis. These findings explain why IR + UV treatment exhibits positive regulation of cell proliferation and enhanced melanin biosynthesis (Erigboz et al., 2023; Subramaniam et al., 2023). Moreover, UV irradiation could increase melanin synthesis through the upregulation of the ERBB2–ERBB4 signaling pathway. As discussed by Ghazi et al. (2023) and based on the process of melanin synthesis, melanin production is accompanied by the generation of hydrogen peroxide and ROS, which are harmful to melanocytes. Without the protection provided by IR, which increases the energy availability of melanocytes, UVB irradiation may further result in melanocyte death.

Investigating the effects of irradiation on DNA damage suggested that IR irradiation could protect melanocytes against DNA fragmentation (Figure 3) and recover the attenuated proliferative activity of melanocytes induced by UV irradiation (Figure 4). These results suggested that IR could potentially provide protection to human melanocytes against DNA fragmentation, apoptosis, and attenuated cell proliferation, which might be a tool to enhance the efficacy of phototherapy.

Prior studies have shown that vitiligo lesions have a lower burden of somatic variation (Gupta et al., 2023) without further biochemical validation of the mechanism. Therefore, this observation leads to two possible open models: (1) the UV shielding model, which suggests that lack of melanin results in less UV absorption, or (2) the apoptotic clearance model, which suggests that UV increases turnover rate in lesional skin. Thus, somatic variations might occur, but the cells are cleared from the tissue before they take any effect. The results obtained from our study support the apoptotic clearance model, in which we observed apoptosis, attenuated proliferation, and DNA fragmentation. Considering that prior studies have shown that vitiligo lesions might be treated through the elimination of ROS (Schallreuter et al., 1999; Tobin et al., 2000), our findings further support the potential of the apoptotic clearance model.

However, there are several limitations to this study. First, the interactions among various cell types within the melanocyte niche were not considered. It is generally recognized that melanocytes interact with surrounding cells in their niche, such as fibroblasts and keratinocytes, to maintain their growth and adhesion. Therefore, it is important to study the behavior of melanocytes in their native environment, e.g., by using human epidermis or engineered epithelial sheets, to understand how cells interact with each other after irradiation with various light sources. Another limitation to this study is that the effect of heat generated by IR was not considered. Although the experiments were carefully conducted, no increase in temperature was detected, and the expression of heat-related genes, such as heat shock proteins, was not upregulated. Considering that IR usually generates heat, it is important to investigate the effect of heat on epidermal melanocytes and determine whether it enhances or counteracts the effects of IR.

## Data Availability

The data can be accessed from China National Center for Bioinformatics with accession number PRJCA071033.
