# Supplementary material for: Infrared radiation alleviates damage to epidermal melanocytes induced by ultraviolet irradiation: potential for improving the efficacy of phototherapy for vitiligo lesions
Source: Front Bioeng Biotechnol. 2026 Aug 21;14:1852981. doi: 10.3389/fbioe.2026.1852981 (PMC13541725; doi:10.3389/fbioe.2026.1852981)
Supplement: Supplementary file 1 [file DataSheet1.pdf]

Supplementary Material

Infrared alleviate damage to epidermal melanocytes induced by ultraviolet irradiation: Potential in improving efficacy of phototherapy for vitiligo lesions

Hua-ye Bao[1], Rong Jin[1], Fuyue Wu[2], Xiaomin Shao[2], Zheng Lin Tan[2*], Aie Xu[1*]

[1] Department of Dermatology, Hangzhou Third People's Hospital, Hangzhou, China

[2] ReMed Regenerative Medicine Clinical Application Institute, Shanghai, People's Republic of China

*** Correspondence:**
Aie Xu (Academic)
xuaiehz@msn.com

Zheng Lin Tan
zhenglin.tan@remed-bio.com

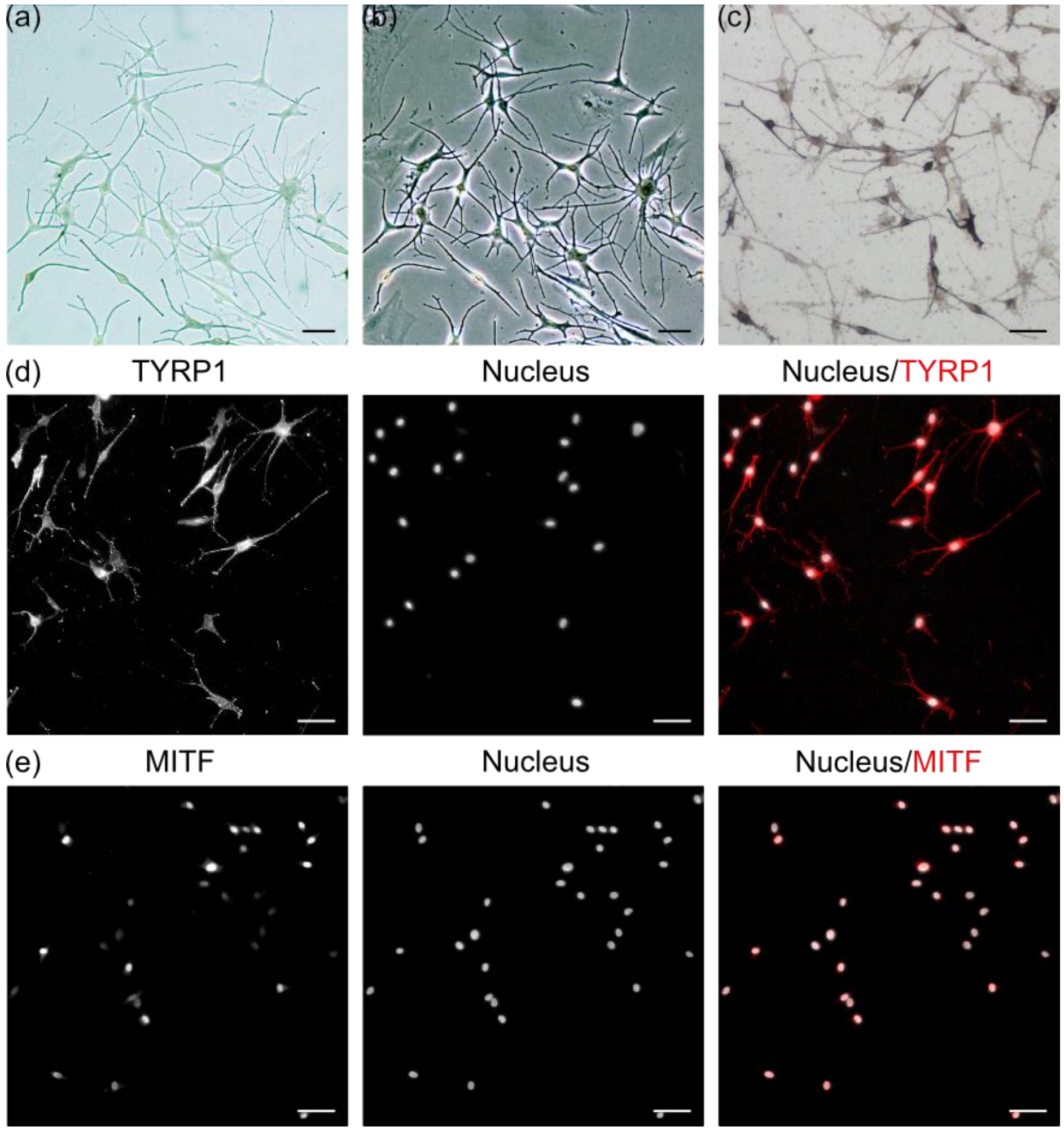

Supplementary Figure 1. Figure 1 Isolation of epidermal melanocytes from human skin. (a) Brightfield photograph of human epidermal melanocytes. (b) Phase contrast photograph of human epidermal melanocytes. (c) *L*-DOPA staining of human epidermal melanocytes. (d) Expression of TYRP1 by human epidermal melanocytes. (e) Expression of MITF by human epidermal melanocytes. The scale bars are 50 μm

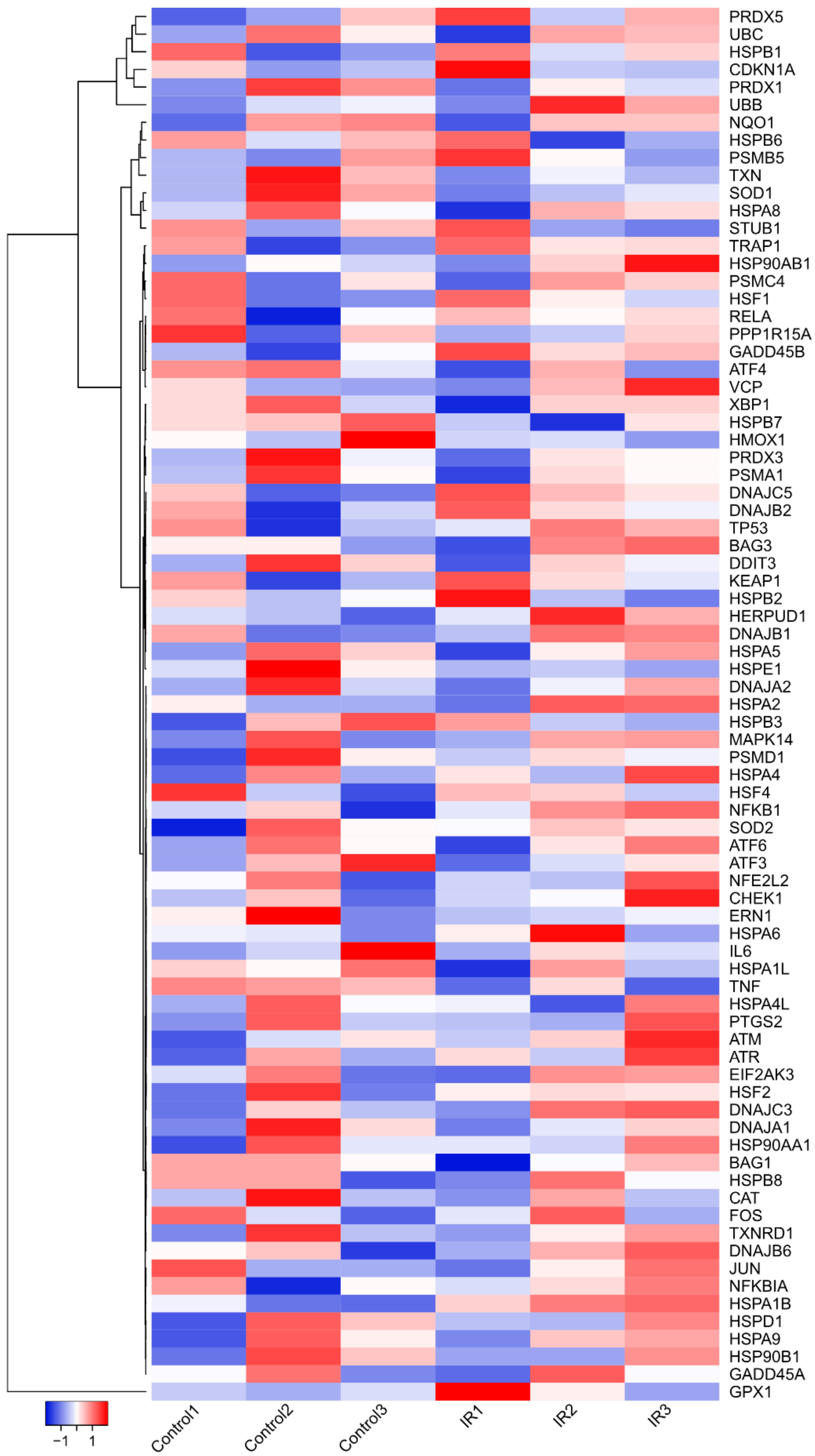

Supplementary Figure 2. Expression of genes related to heat response after IR irradiation. No significant changes in genes expression related to heat response was detected in melanocytes after IR irradiation.
